# *Strongylocentrotus* *nudos* Egg Polysaccharide induces autophagy and apoptosis in leukaemia cells by regulating mitochondrial function

**DOI:** 10.1111/jcmm.15995

**Published:** 2020-12-01

**Authors:** Chong Wang, Mengya Li, Lingling Li, Xiaohui Shen, Yanfang Liu, Shujuan Wang

**Affiliations:** ^1^ Department of Hematology The First Affiliated Hospital of Zhengzhou University Zhengzhou China

**Keywords:** apoptosis, autophagy, leukaemia, mitochondria, NF‐κB, SEP

## Abstract

In this study, we investigated the ability of the Polysaccharide from the Eggs of *Strongylocentrotus nudus* (SEP) to regulate cellular autophagy and apoptosis in leukaemia cells. Human acute myeloid leukaemia (AML) cells (HL60) and murine AML cells (L1210) treated with SEP were used to assess viability using Cell Counting Kit‐8, cytotoxicity by measuring lactate dehydrogenase release, the generation of reactive oxygen species (ROS) by DCFH‐DA staining. In addition, we utilized a mouse model of leukaemia in which L1210 cells were injected into DBA/2 mice by sub‐axillary injection. Treatment with SEP decreased cell viability, increased in cytotoxicity and increased the release of ROS in a dose‐dependent manner. SEP treatment was also associated with the activation of pro‐apoptotic proteins cleaved caspase‐3, cleaved caspase‐9 and cleaved poly (ADP‐ribose) polymerase (PARP). Activation of the apoptotic pathway led to the release of cytochrome C (CytoC) into the cytosol of the cell resulting in decreased membrane potential. The effect of SEP treatment was depended on the activation of the nuclear factor kappa‐B (NF‐κB) signalling pathway as SEP treatment led to an increase in NF‐κB phosphorylation, and inhibition of NF‐κB signalling using PDTC blocked SEP‐mediated activation of apoptosis. Treatment with SEP also prolonged survival time in our leukaemia mouse model and was associated with diminished tumour volume, increased leucocyte and lymphocyte proliferation, promoted pro‐inflammatory factor release in serum and enhanced immune function. Taken together, these data suggest that SEP inhibits the progression of leukaemia by initiating mitochondrial dysfunction, autophagy, and apoptosis *via* the NF‐κB signalling pathway.

## 1 INTRODUCTION

Acute myeloid leukaemia (AML) originates in hematopoietic stem cells, and is characterized by leukaemia cell proliferation and infiltration as well as fever, infection and bleeding.^1^ Normal hematopoiesis is hindered due to the increased proliferation of primitive myeloid cells in the bone marrow while differentiation and apoptosis are suppressed.^2^ Moreover, AML has a high mortality and recurrence rate, which makes AML the most common haematological malignancy.^3^ Recent progress in identifying anti‐apoptotic pathways in AML primary cells has led to the development of several new drugs currently moving through the drug development pipeline, either as individual therapies or in combination with conventional chemotherapy regimens.^4^ Nevertheless, there is still significant need for the identification of additional effective treatments for AML.

Cells initiate the process of autophagy in response to stressful situations, such as nutrient deprivation, as a means of self‐preservation. During autophagy, the cell begins to breakdown non‐essential molecules to prolong cell survival. When cells have exhausted superfluous proteins, the cell may initiate apoptosis or cell death.^5^ Autophagy plays an important role in regulating the growth, differentiation and death of cells.^6^ AML cells display limited myeloid differentiation, inhibition of apoptosis in progenitor cells and enhanced self‐renewal capacity.^7^ As autophagy promotes AML cell survival, targeting autophagy in leukaemia cells may provide new methods for the treatment of AML.

Several polysaccharides exhibit anti‐tumour effects by changing the biochemical properties of cell membranes, inducing tumour cell differentiation and apoptosis, or affecting signal transmission between tumour cells.^8^ Several studies have documented the ability of exogenous polysaccharides to alter the biology of normal organisms as well as exert beneficial therapeutic effects in diseased organisms.^9^ At present, hundreds of polysaccharides are known to have definitive drug effects.^10^ *Strongylocentrotus nudus* egg polysaccharide (SEP) is a water‐soluble polysaccharide isolated from the eggs of *Strongylocentrotus nudus*.^11^ Preliminary in vitro experiments show that SEP can significantly promote the proliferation of splenic lymphocytes.^12^ In addition, SEP inhibits tumour growth by affecting telomerase activity and angiogenesis.^12^, ^13^ To date, few studies have investigated a relation between SEP and AML. We used SEP to treat human and mouse AML cell lines and study its effect on proliferation, apoptosis, creation of reactive oxygen species, markers of autophagy, and in vivo tumour growth. Because the nuclear factor kappa‐B (NF‐κB) pathway plays an important role in the occurrence of AML, we studied the impact of SEP treatment on the regulation of immune and inflammatory responses.^14^ While the role of NF‐κB in the progression of AML is not fully understood, NF‐κB is constitutively expressed in AML cells and leukaemia stem cells in many patients.^15^ Our study identifies a mechanism by which SEP regulates AML autophagy‐related phenomena and may represent a potential therapeutic in the treatment of AML.

## 2 MATERIALS AND METHODS

### 2.1 Polysaccharide extraction and cell culture

*Strongylocentrotus nudus* egg polysaccharide was extracted from *Strongylocentrotus nudus* eggs and analysed by high‐performance size‐exclusion chromatography, which showed a single‐symmetrical spike with a purity of 98.8%. The human AML cell line, HL‐60 (CCL‐240), and murine AML cell line, L1210 (CCL‐219), were both purchased from ATCC (Manassas, VA, USA) and cultured according to the ATCC’s instructions. In brief, HL‐60 and L1210 cells were cultured in high‐glucose dulbecco's modified eagle medium (D5796, Sigma‐Aldrich, St Louis, MO, USA) supplemented with 15% foetal bovine serum (F8687, Sigma‐Aldrich), 100 Unit/mL penicillin (I9532, Sigma‐Aldrich) and 100 mg/mL streptomycin (85886, Sigma‐Aldrich) in a humidified cell culture incubator containing 5% CO_2_ at 37°C.

### 2.2 Cell viability

Cell Counting Kit‐8 (CCK‐8) (C0038, Beyotime Biotechnology Co., Ltd., Shanghai, China) was utilized to measure cell viability. Leukaemia cells were seeded into 96‐well plates with a final volume of 100 μL culture medium/well. After 12‐hour culture, fresh medium containing 50, 100 or 200 μg/mL of SEP was added and the same volume of medium without SEP was used as the background control. After SEP was added, 10 μL/well CCK‐8 solution was also added to plates and the plates were allowed to incubate for 72 hours. After additional 4 hours incubation at 37°C, each well was examined for absorbance at 450 nm using a 96‐well plate reader (ThermoFisher Scientific, Waltham, MA, USA). Eight repeated experiments were used for each group.

### 2.3 Detection of intracellular reactive oxygen species (ROS) using fluorescence spectroscopy

The intracellular production of ROS was measured utilizing the dichloro‐dihydro‐fluorescein diacetate (DCFH‐DA) dye assay. With oxidant, DCFH was transformed into fluorescent 2,7‐dichlorofluorescein (DCF). Cells were incubated with 50, 100 or 200 μg/mL of SEP for 24h, followed by 10 μmol/L DCFH‐DA treatment for 30 minutes. Fluorescence intensity was evaluated using a fluorescence microplate reader (Thermo Fisher Scientific, Waltham, MA, USA) at an excitation wavelength of 485 nm and an emission wavelength of 530 nm.^16^

### 2.4 Cytotoxicity as measured by Lactate dehydrogenase (LDH) release

The amount of LDH released by cells was detected using the Cytotoxicity Detection Kit (C0016, Beyotime). Cells were treated with 50, 100 or 200 μg/mL of SEP for 24 hours, centrifuged at 400 *g* for 5 minutes and the supernatant was removed. The LDH release reagent was diluted 1:10 in phosphate buffer saline (PBS) and then added to the pellet. One hour later, samples were centrifuged and 120 μL supernatant per well was transferred to a new plate and the plate was read on a plate reader (Thermo Fisher Scientific, Waltham, MA, USA) at an absorbance of 490 nm.

### 2.5 Flow cytometry

Initiation of apoptosis in leukaemia cells was tested using the Annexin V‐fluorescein isothiocyanate (FITC)/propidium iodide (PI) apoptosis kit (C1062L, Beyotime) and measuring staining using flow cytometry. Briefly, cells were detached with trypsin and then centrifuged at 100 *g*, for 5 minutes at room temperature and the residual medium and trypsin was removed. Afterwards, 50, 100 or 200 μg/mL of SEP were added to cells and they were allowed to incubate for 24 hours. Cells were harvested the following day and the cell pellet was washed twice with PBS and resuspended in 500 μL loading buffer. FITC‐Annexin V and PI were then added to the cells (5 μL and 10 μL, respectively) in dark at room temperature for 5 minutes, immediately followed by flow cytometry analysis in which the FITC signal channel (FL1) and the PI signal channel (FL2) were used to detect signal (Beckman, Brea, CA, USA). Each sample was assessed by determining the fraction of 1 × 10^4^ cells present in one of the following 4 groups: (a) viable cells (Annexin V−/PI−, Q3), (b) early apoptotic cells (Annexin V+/PI−, Q4), (c) late apoptotic cells (Annexin V+/PI+, Q2) and (d) dead cells (Annexin V−/PI +, Q1). Both of the percentage of early apoptotic cells (Q4) and late apoptotic cells (Q2) were included in the total apoptotic cell count.^17^

### 2.6 Evaluating anti‐tumour effects in vivo

All experiments involving mice were conducted according to the National Institutes of Health standards and guidelines and approved by the Experimental Animal Ethics Committee.^18^ DBA/2 mice with an average weight of 20.0 ± 2.0 g were purchased from the Animal Experiment Research Center of the Zhejiang Chinese Medical University (Hangzhou, Zhejiang, China). All mice were maintained in the animal facility for at least 2 weeks before each experiment. Mice were randomly caged in clear plastic cages containing wood shavings for bedding at 25 ± 2°C with a 12‐hour light/dark cycle, receiving standard laboratory feed, ad libitum. We injected 1 × 10^6^ L1210 cells subcutaneously into the sub‐axillary region of each mouse.^19^ The day after injection of AML cells, the mice were injected with either saline (negative control group), 20 mg/kg cyclophosphamide (CTX) (CTX‐treated group) or SEP at a 10 or 40 mg/kg. Ten days after inoculation, all mice were anaesthetized with 3% pentobarbital sodium and weighed. Blood samples were harvested via cardiac puncture before mice were killed. The animals were observed for mortality throughout the treatment period and mean survival time (days) were calculated using the following formula: [(TC)/C] × 100％, where T was the number of survival days of the treated groups and C was the survival days of the control group.

### 2.7 Leucocyte and lymphocyte proliferation assay

Murine blood samples were collected and held on ice until the leucocyte and lymphocyte cell counts could be analysed using an auto blood analyzer (CA620‐VET, Sweden). Briefly, double distilled water was used as the blank control and a standard reference material as a quality control. The blood samples were shaken gently and placed under needle to measure leucocyte and lymphocyte cell counts.^20^

### 2.8 Enzyme‐linked immunosorbent assay (ELISA) of TNF‐α, IL‐6 and IFN‐ γ

Tumour necrosis factor‐α (TNF‐α), interleukin‐6 (IL‐6) and interferon‐γ (IFN‐γ) levels in plasma or cells were measured using ELISA (Beyotime). Briefly, the samples were added to an immunoplate coated with TNF‐α, IL‐6 or IFN‐γ and incubated at 37°C for 30 minutes. Plates were washed five times with PBS and enzyme‐labelled secondary antibody was added. The microplate was washed after 30‐min incubation at 37°C, and then substrate solution was added and the plates were incubated for another 30 minutes at 37°C in dark. The reaction was terminated with H_2_SO_4_ and readings were taken for each well at an absorbance of 450 nm with the plate reader (ThermoFisher Scientific, Waltham, MA, USA). A standard curve was plotted using known concentrations of the cytokine of interest and absorption readings taken during the assay. TNF‐α, IL‐6 and IFN‐γ levels in each sample were calculated according to the standard curve.^20^

### 2.9 Microarray analysis

L1210 cells were seeded in 100 mm cell culture dishes (1 × 10^7^/dish) and cultured overnight. Cells were removed from the plates and centrifuged at 250 *g* for 5 minutes. After removing the supernatant, the cells were treated with 100 μg/mL SEP treatment for 24 hours. The NC samples were treated with a volume of medium equal to that used for the SEP‐treated samples. Total RNA was extracted from 5 × 10^6^ cells and purified by phenol‐chloroform‐isoamyl alcohol extraction (25:24:1). Spectrophotometry and capillary electrophoresis (Agilent 2100 Bioanalyzer, USA) were utilized to confirm RNA purity using the A260/A280 ratio. RNA was processed according to the Affymetrix's instructions and hybridized to Affymetrix HG‐U133A oligonucleotide microarrays. Raw data were processed by Microarray Suite 5.0. All probe scales were set to a constant value of 500 for each microarray. Principal component analysis (PCA) was adopted to analyse the gene profiles of the control and SEP‐treated group using Cluster 3.0. We selected genes with a fold change of at least 2 between the two groups for further analysis and these genes were subjected to Gene Ontology (GO) analysis and KEGG functional annotation using DAVIE database (https://david.ncifcrf.gov/home.jsp). Visualization was performed by using R and Cytoscape.^21^

### 2.10 Western blot analysis

For Western blot analysis, untreated and SEP‐treated (50, 100 and 200 μg/mL) L1210 cells were lysed using Radio Immunoprecipitation Assay buffer (Generay, Shanghai, China) containing protease inhibitor (87786, Thermo Fisher Scientific, Waltham, MA, USA). In some experiments, L1210 cells were incubated with 10 μmol/L pyrrolidine dithiocarbamate (PDTC) (S1809, Beyotime), a specific inhibitor of the NF‐κB pathway, for 1h, before treatment with SEP (200 μg/mL) for 24h. Lysates were centrifuged at 7000 *g* for 10 minutes at 4°C. We then collected the supernatant and measured the protein concentration by bicinchonininc acid kit (23227, Thermo Fisher Scientific, Waltham, MA, USA). The proteins were subsequently denatured and resolved using sodium dodecyl sulphate polyacrylamide gel electrophoresis, followed by electrophoretic transfer to polyvinylidene fluoride membranes (Millipore, Billerica, MA, USA). Membranes were subsequently incubated with the following primary antibodies diluted in buffer and incubated overnight at 4°C: rabbit anti‐cleaved caspase‐3 (ab2302, 1:500), rabbit anti‐cleaved caspase‐9 (ab2324, 1:200), rabbit anti‐cleaved PARP (ab32064, 1:1000), mouse antibody to light chain 3‐II (LC3‐II) (ab51520, 1:1000), mouse antibody to Beclin1 (BECN1) (ab114071, 1:1000) all from Abcam (Cambridge, MA, USA), rabbit anti‐phospho‐NF‐κB p65 (Ser536) (#3033, 1:1000), rabbit anti‐NF‐κB p65 (#8242, 1:1000) and rabbit anti‐β‐actin (#4970, 1:1000) from CST (Danvers, MA, USA). The next day, membranes were incubated with secondary antibody for 1‐2 hours at room temperature. In parallel experiments, cytosol and mitochondria were separated by cytosol/mitochondria fractionation kit (Calbiochem, USA) according to the manufacturer's instructions. Rabbit anti‐Cytochrome C (ab133504, 1:5000, Abcam) and rabbit anti‐cytochrome oxidase IV (COX IV) (ab202554, 1:2000, Abcam) primary antibodies were used to probe membranes containing cytosol/mitochondria samples followed by incubation with secondary antibody conjugated to horseradish peroxidase overnight at 4°C. After washing, enhanced chemiluminescence (35050, Thermo Fisher Scientific, Waltham, MA, USA) was added to the membrane, and signal was subsequently detected using the ChemiDoc™ XRS imaging system (Bio‐Rad, Hercules, CA, USA). Protein bands were analysed using Quantity One software (Bio‐Rad).^22^

### 2.11 Mitochondrial membrane potential measurement (ΔΨm)

The mitochondrial membrane potential of L1210 cells was assessed by measuring the potential‐dependent accumulation of 5,5’,6,6’‐tetrachloro‐1,1’,3,3’‐tetraethyl benzimidazolyl carbocyanine iodide (JC‐1) in the mitochondria (C2006, Beyotime). Cells were seeded onto 24‐well plates at a density of 1 × 10^5^ cells per well. After SEP treatment for 24 hours, cells were stained with 5 μmol/L JC‐1 dye for 20 minutes at 37°C, and then washed twice with cold PBS followed by immediately reading on a fluorescence microscope (Leica DM3000B, Mannheim, Germany). The excitation and emission wavelengths of the JC‐1 monomer and polymer were 490 nm/530 nm and 525 nm/590 nm, respectively. Mitochondrial membrane potential changes were calculated by the red to green fluorescence ratio.^16^

### 2.12 Reverse transcription‐quantitative polymerase chain reaction (RT‐qPCR)

Total RNA was extracted using TRIzol (Invitrogen Life Technologies, Carlsbad, CA, USA), and the quality was tested using agarose gel electrophoresis. RNA was considered intact and sufficient for use in subsequent experiments if there were three clear bands (28S, 18S and 5S) and the 28S band was approximately twice as strong as the 18S band. The RNA concentration was quantified using the Nanodrop 2000 (Thermo Fisher Scientific, Waltham, MA, USA). According to the manufacturer's protocols, 1 μg total RNA was reverse transcribed into cDNA using 5 × g DNA Eraser Buffer and gDNA Eraser and the following thermocycler conditions: 42°C for 2 minutes, then 37°C for 15 minutes and then 85°C for 5 seconds. Then, gene expression was quantified by RT‐qPCR using the SYBR^®^ Premix Ex TaqTM (Tli RNaseH Plus) kit (RR820A, TaKaRa, Japan) with the ABI7500 real‐time PCR instrument (Thermo Fisher Scientific, Waltham, MA, USA). The reaction conditions were as follows: at 95°C for 10 minutes followed by 40 PCR cycles of 95°C for 15 seconds and 60°C for 30 seconds. The target mRNA level was normalized to glyceraldehyde‐3‐phosphate dehydrogenase (GAPDH). Fold change of gene was calculated by using 2^−△△CT^ method: ΔΔCT = Ct (treated) – Ct (control), ΔCT = Ct (target gene) – Ct (GAPDH). The Ct refers to the amplification cycle number when the fluorescence intensity reaches a set threshold, during a period of logarithmic amplification. mRNA primers were synthesized by Sangon Biotech (Shanghai, China), and their sequences are shown in Table 1. All experiments were repeated three times with three triplicates for each variant.^23^, ^24^, ^25^

**TABLE 1:** Primer sequences for RT‐qPCR

Gene	Sequence
ATG3	F: 5'‐AGGAATCAAAATTTAAGGAAA‐3'
	R: 5'‐TTTGTCTGTCGGAAGATATG‐3
ATG5	F: 5'‐AGCAGCTCTGGATGGGACTGC‐3'
	R: 5'‐GCCGCTCCGTCGTGGTCTGA‐3'
ATG7	F: 5'‐GCTCCTCATCACTTTTTGCCAACA‐3'
	R: 5'‐GGAGCCACCACATCATTGC‐3'
ATG12	F: 5'‐GGAGACACTCCTATAATGAAA‐3'
	R: 5'‐ATAAATAAACAACTGTTCCGA‐3'
BECN1	F: 5'‐GAATGCTGTTTGATACTGTG‐3'
	R: 5'‐TTTTAAGGAAAAACATACAGG‐3'
GAPDH	F: 5'‐TGCCAAGTATGATGAACATCAAGAAG‐3'
	R: 5'‐GGTCCTCAGTGTAGCCCAAGAT‐3'

Abbreviations: ATG, autophagy‐related gene; BECN1, Beclin1; F, forward; GAPDH, glyceraldehyde‐3‐phosphate dehydrogenase; R, reverse; RT‐qPCR, reverse transcription‐quantitative polymerase chain reaction.

### 2.13 Statistical analysis

All data are shown as the mean ± standard deviation and analysed with SPSS 21.0 software (IBM, Armonk, NY, USA). Statistical comparisons were performed using unpaired *t* test between two groups and one‐way analysis of variance (ANOVA) followed by Tukey's post hoc analysis for comparison of more than two groups. Survival analysis was performed using Kaplan‐Meier and log‐rank analysis. For all experiments, *P* < .05 was considered statistically significant.

## 3 RESULTS

### 3.1 SEP promotes apoptosis and prevents leukaemia cell proliferation

**FIGURE 1:**
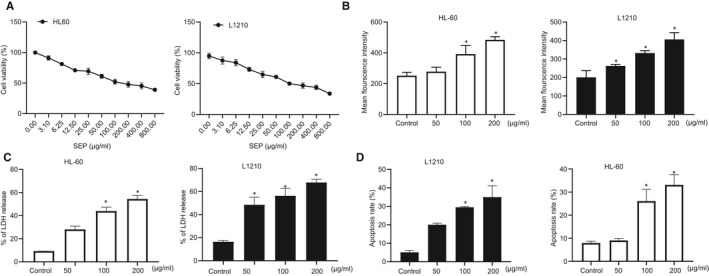
SEP inhibited cell growth and induced apoptosis in leukaemia cell lines. A, CCK‐8 measurement of L1210 and HL‐60 cell viability. B, Detection of ROS in L1210 and HL‐60 cells using DCFH‐DA fluorescent probe labelling. C, Measurement of LDH activity as an indicator of cytotoxicity in L1210 and HL‐60 cells. D, Apoptosis rates of L1210 and HL‐60 cells using flow cytometry. Experiments represent three or more replicates. Data are shown as mean ± standard deviation. Comparisons between multiple groups were conducted using one‐way ANOVA followed by Tukey's post hoc analysis. * indicates *P* < .05 compared with the control group

SEP is thought to suppress tumorigenesis by enhancing immune function, but the underlying mechanism is unclear.^11^, ^12^, ^13^, ^26^, ^27^ Previous studies indicated that SEP may stimulate T lymphocyte^11^ and macrophage function,^27^ as well as up‐regulate IL‐2, TNF‐α and IFN‐γ expression.^13^, ^26^ The CCK‐8 assay was utilized to measure the effects of SEP on the cell growth and viability of HL60 and L1210 cells. Increasing concentrations of SEP progressively reduced cell viability of L1210 cells (Figure 1A). Furthermore, treatment with SEP also led to dose‐dependent increases in intracellular ROS levels compared with untreated cells (Figure 1B). Measurement of lactate dehydrogenase (LDH) as a marker of cytotoxicity indicated that there was more cell death in SEP‐treated AML cells than in untreated cells (Figure 1C). Annexin V and Propidium iodide staining followed by flow cytometry also suggested that SEP promoted apoptosis in SEP‐treated AML cell lines (Figure 1D). Taken together, these results indicate that SEP inhibited leukaemia cell growth and induced apoptosis in leukaemia cells.

### 3.2 SEP modulated immune responses to prolong the survival of leukaemic mice

**FIGURE 2:**
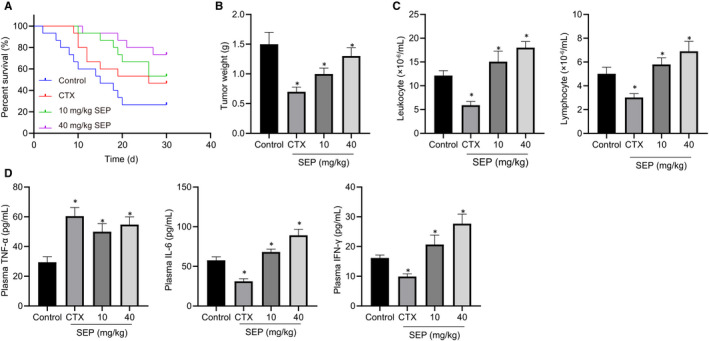
SEP extended survival in a mouse model of leukaemia by enhancing immune function. A, Survival time of the mice that received control, CTX or SEP treatment followed by inoculation with L1210 cells (n = 15). B, The ratio of tumour weight to mouse weight for each treatment group (n = 10). C, Quantitation of leucocytes and lymphocytes (n = 10). D, ELISA analysis of TNF‐α, IL‐6 and IFN‐γ for each treatment group. Experiments represent three or more replicates. Data are shown as mean ± standard deviation. Comparisons between multiple groups were conducted using one‐way ANOVA followed by Tukey's post hoc analysis. * indicates *P* < .05 compared with the control group

To demonstrate the anti‐leukaemic effects of SEP in vivo, we inoculated DBA/2 mice with L1210 cells by sub‐axillary injection.^28^ As CTX is a chemotherapeutic agent widely used in the clinical treatment of leukaemia, it was chosen to serve as a positive control in our mouse model.^29^ When the survival curves associated with each treatment group were compared, treatment with SEP extended the survival time of leukaemic mice in a dose‐dependent fashion (Figure 2A). Notably, compared with control group, both CTX and SEP significantly reduced tumour size in vivo (Figure 2B). Furthermore, SEP promoted the proliferation of murine leucocytes and lymphocytes after inoculation with L1210 cells (Figure 2C). Several cytokines are known to regulate tumour progression, including IL‐6, TNF‐α and IFN‐γ.^30^ ELISA analysis of IL‐6, TNF‐α and IFN‐γ in the serum of tumour‐bearing mice revealed a significant increase in their concentration after SEP treatment compared with NCs (Figure 2D). These data suggest that SEP prolongs the lifespan of leukaemia‐bearing mice by enhancing anti‐tumour immunity in vivo.

### 3.3 Identification of SEP‐regulated Pathways using Microarray analysis

**FIGURE 3:**
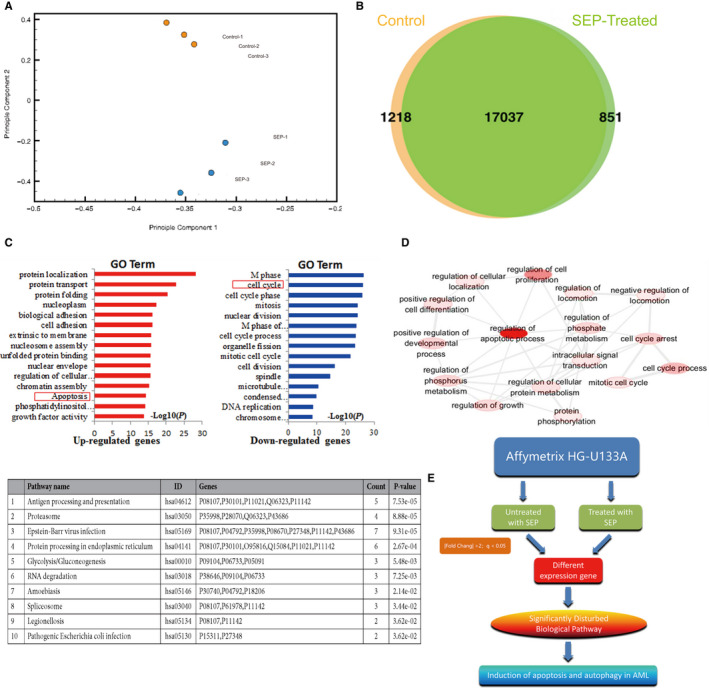
Microarray analysis of differentially expressed genes. A, PCA analysis scatter plot depicting the degree of dispersion between genes from control samples and SEP‐treated samples. B, Comparison of differential gene expression between control and SEP‐treated samples. C, GO analysis of differentially expressed genes. D, Predicted network of interacting genes based on GO and KEGG analysis. E, Flowchart of microarray and informatics analysis

We compared the transcriptomes of leukaemia cells that were or were not treated with SEP using the GeneChip Microarray assay. PCA analysis showed significant segregation of SEP‐treated and non‐treated samples (Figure 3A), and a Venn diagram was created to illustrate any overlap of differentially regulated genes (Figure 3B). GO and KEGG pathway analysis using the DAVID database were carried out to identify the potential biological functions of the differentially expressed genes (Figure 3C). The results suggest that SEP treatment primarily affects cell cycle, DNA replication and cell apoptosis‐related biological processes. In addition, KEGG pathway analysis also identified adhesion and mitotic nuclear division as being regulated by SEP. In L1210 cells, SEP activated apoptosis and adhesion and regulated cell survival, immunity and metabolism. Analysis of overlapping pathways by GO and KEGG analysis identified pro‐apoptotic and anti‐tumour functions of SEP.

### 3.4 SEP induces ROS‐mediated mitochondrial apoptosis by activating the NF‐κB signalling pathway

**FIGURE 4:**
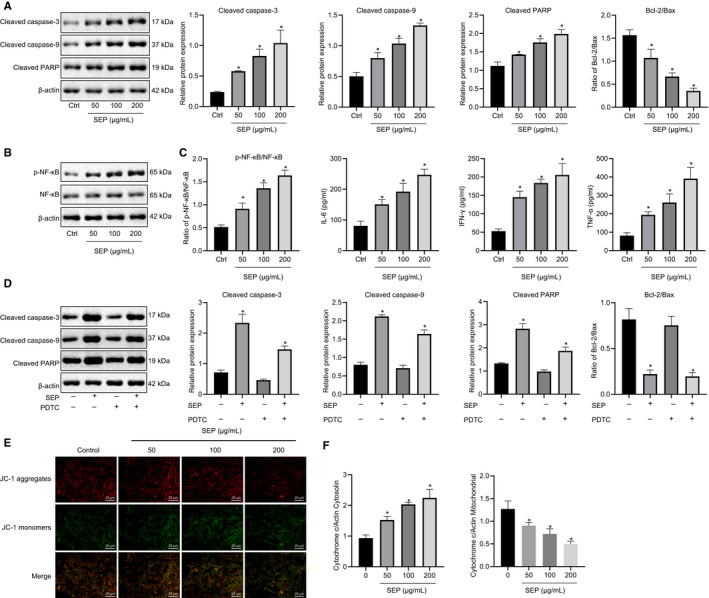
SEP induces apoptosis in L1210 cells via interfering mitochondrial function. A, Western blot analysis of cleaved caspase‐3, cleaved caspase‐9 and cleaved PARP after 24 h treatment with SEP compared to untreated control. B, Western blot analysis of p‐NF‐κB and NF‐κB in L1210 cells after treatment with SEP at various concentrations. C, ELISA analysis of TNF‐α, IL‐6 and IFN‐γ expression in L1210 cells after treatment with SEP at various concentrations for 24 h. D, Western blot analysis of cleaved caspase‐3, cleaved caspase‐9 and cleaved PARP after treatment with SEP (200 µg/mL) and with or without the addition of NF‐κB inhibitor PDTC for 24 h. E, L1210 cells were treated with SEP at various concentrations for 24 h, stained with JC‐1 dye and observed with an epifluorescent microscope. Scale bar = 25 μm. F, L1210 cells were treated with SEP at various concentrations for 24 h, subjected to mitochondrial and cytoplasmic separation, and the CytoC content in the mitochondria and cytosol was measured by Western blot. Experiments represent three or more replicates. Data are shown as mean ± standard deviation. Comparisons between multiple groups were conducted using one‐way ANOVA followed by Tukey's post hoc analysis. * indicates *P* < .05 compared with the control group

Next, we investigated the activation of apoptosis. B‐cell lymphoma‐2 (Bcl‐2) associated protein X (Bax) and Bak are two Bcl‐2 family members, which promote apoptosis by causing the permeabilization of the outer mitochondrial membrane and the release of apoptotic‐related factors into the cytosol, including cytochrome c (CytoC).^31^ CytoC then combines with Apaf‐1 to form the caspase‐9‐activating apoptosome.^32^ Because treatment of leukaemia cells with SEP led to a 50% decrease in cell viability by 24 hours (Figure S1), this time point was selected for all subsequent experiments. Western blot analysis was performed in L1210 cells to detect expression levels of cleaved caspase‐3, and caspase‐9 as well as caspase‐3 substrate poly (ADP‐ribose) polymerase (PARP). Our results showed a dose‐dependent increase in cleaved caspase‐3, caspase‐9, and PARP after SEP treatment (Figure 4A). SEP also increased the phosphorylation of NF‐κB p65 (Figure 4B) and the expression of inflammatory cytokines TNF‐α, IL‐6 and IFN‐γ (Figure 4C). In addition, the effect of SEP on cleaved caspase‐3, caspase‐9 and PARP expression was eliminated by addition of PDTC, a specific inhibitor of the NF‐κB pathway (Figure 4D). SEP treatment led to a decrease in mitochondrial membrane potential (Figure 4E), an increase in CytoC released into the cytosol and a concomitant reduction in CytoC in mitochondria (Figure 4F). Similar results were observed in the human AML cell line, HL‐60 (Figure S2). SEP treatment stimulated the release of TNF‐α, IL‐6 and IFN‐γ and increased mitochondrial membrane potential, which were suppressed by the addition of NF‐κB inhibitor (Figure S3). In summary, SEP treatment lowered mitochondrial membrane potential, triggered the release of mitochondrial CytoC, and activated caspase‐3 and caspase‐9 promoting apoptosis and that SEP function was dependent on the NF‐κB signal pathway.

### 3.5 SEP regulates autophagy to promote leukaemia cell survival

**FIGURE 5:**
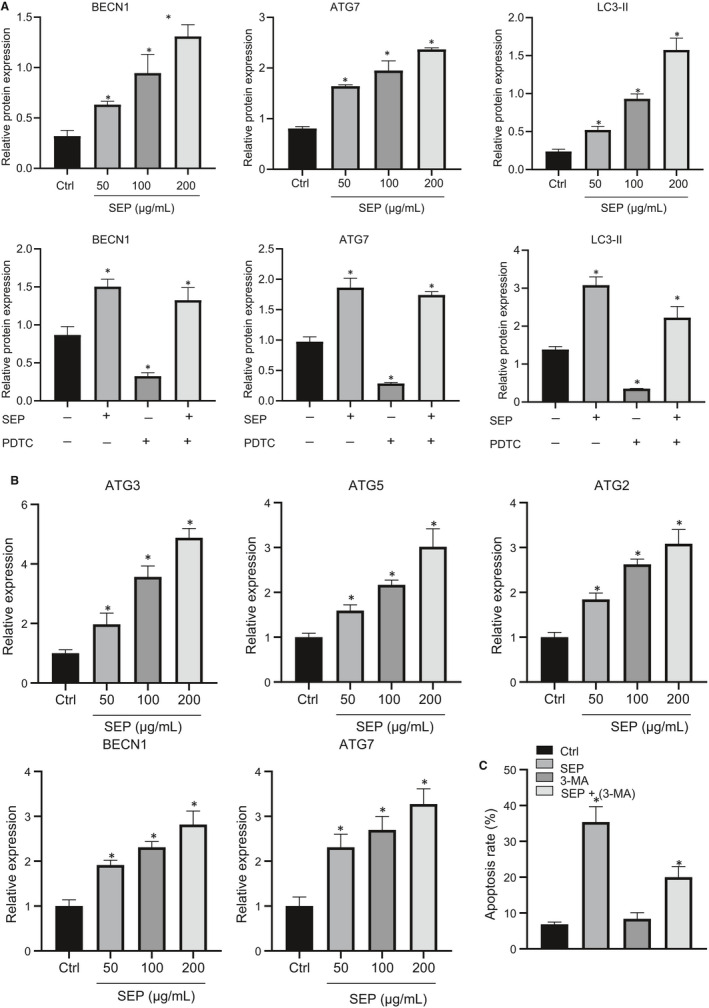
SEP‐induced L1210 cell apoptosis through NF‐κB‐dependent autophagy. A, L1210 cells were treated with various concentrations of SEP for 24 h, or SEP (200 μg/mL) with/without NF‐κB inhibitor PDTC followed by analysis of LC3‐II and BECN1 expression using Western blot analysis. B, L1210 cells were treated with different concentrations of SEP (50, 100 and 200 μg/mL) followed by extraction of total RNA. The mRNA levels of ATG3, ATG5, ATG7, ATG12 and BECN1 were measured by RT‐qPCR. C, L1210 cells were incubated with SEP (200 μg/mL) with/without autophagy inhibitor 3‐MA (2 mmol/L), followed by flow cytometry with annexin V and PI staining. Experiments represent three or more replicates. Data are shown as mean ± standard deviation. Comparisons between multiple groups were conducted using one‐way ANOVA followed by Tukey's post hoc analysis. * indicates *P* < .05 compared with the control group

The connection between autophagy and apoptosis is still not well understood. Autophagy is a well‐studied process, which regulates cytoplasm and organelle turnover. Cells use autophagy as a survival mechanism during extreme conditions such as nutrient deprivation, but it may also be involved in non‐apoptotic cellular death pathways.^33^ Previous reports suggest that dexamethasone treatment induces apoptosis in leukaemia cells by activating acute promyelocytic leukaemia protein and Akt‐dependent autophagy leading to mitochondrial dysfunction.^34^ As SEP‐induced mitochondrial dysfunction was the key cause of leukaemia cell apoptosis, we decided to investigate whether SEP promoted leukaemia cell apoptosis by activating autophagy. Treatment of murine AML L1210 cells with SEP led to concentration‐dependent increases in LC3‐II and BECN1 expression, demonstrating that SEP could activate the autophagy pathway (Figure 5A, upper). Activation of autophagy was blocked by treatment with the NF‐κB inhibitor PDTC (Figure 5A, lower). Our results revealed that activation of the NF‐κB pathway was required for SEP‐mediated autophagy in leukaemia cells. RT‐qPCR analysis showed that autophagy‐related gene 3 (ATG3), ATG5, ATG7, ATG12 and BECN1 were also up‐regulated by SEP treatment in a dose‐dependent manner (Figure 5B). Treatment with 3‐methyladenine (3‐MA) to block autophagy was able to prevent SEP‐induced leukaemia cell apoptosis (Figure 5C). Similar results were observed in the human AML cell line, HL‐60 (Figure S4). Cumulatively, our data suggest that the SEP induction of apoptosis is mediated by NF‐κB activation of autophagy.

## 4 DISCUSSION

This study examined the ability of SEP to promote apoptosis in leukaemia cells. We utilized a leukaemia mouse model in which DBA/2 mice received sub‐axillary injection of L1210 cells to study the effect of SEP treatment on the survival of mice with AML. After treatment with SEP, the mice were evaluated for their expression of pro‐inflammatory cytokines TNF‐α, IL‐2 and IFN‐γ in the serum of mice. Our results indicated that SEP could inhibit the proliferation of leukaemia cells, which was consistent with the reported role of SEP in other tumour cells.^12^ Furthermore, we showed that SEP had a concentration‐dependent inhibitory effect on leukaemia progression.

Apoptosis, or programmed cell death, is the process whereby cells undergo a controlled self‐destruction in response to the activation of specific endogenous or exogenous signals, and under the control of a family of related genes.^35^ Caspase‐3 is a key enzyme involved in the mitochondrial activation of apoptosis.^36^, ^37^, ^38^ Our results showed that as the concentration of SEP increased caspase‐3 enzyme activity, suggesting that the activation of caspase‐3 played an important role in activating the apoptotic pathway in leukaemic cells after SEP treatment.

Our study further found that SEP‐induced leukaemia cell apoptosis through the NF‐κB signalling pathway. Specifically, SEP promoted the activation of NF‐κB leading to an increase in markers associated with autophagy. NF‐κB is known to be an important inhibitor of apoptosis as well, promoting self‐protection and adaptation, and is itself a target in the treatment of various cancers and other proliferative diseases.^39^ In addition, NF‐κB can inhibit cellular autophagy by activating the mammalian target of rapamycin (mTOR) pathway.^40^ NF‐κB is also known for its ability to regulate the expression of a variety of cytokines, growth factors and other basic cellular messenger proteins, allowing it to regulate a variety of cell functions.^41^ Inhibition of NF‐κB activity can promote tumour cell apoptosis.^42^ Our results suggest SEP promotes mitochondrial dysfunction and apoptosis *via* activation of the NF‐κB pathway.

Mitochondria are one of the most sensitive organelles to various injuries.^43^ When cells are stimulated, the number, size and structure of mitochondria can undergo pathological changes, affecting the survival of cells.^44^ Mitochondrial‐mediated regulation of autophagy has both a direct and indirect relationship with the onset of tumours and represents a key regulator of quality control in tumour cells. The outcome of activation of the autophagy pathway may differ depending on the stage of tumour development.^45^ In this experiment, the relationship between NF‐κB and the activation of autophagy was examined by investigating the activation of LC3‐II protein. NF‐κB activation led to increases in LC3 and activation of autophagy.

Moreover, this study found that SEP prolonged the lifespan of mice with leukaemia by enhancing the immune system function. Similar to SEP, APS‐1II extracted with NaOH can activate peripheral blood leucocytes and lymphocytes, stimulate the secretion of cytokines TNF‐α, IFN‐γ and IL‐2, promote the proliferation of splenocytes, enhance the phagocytic activity of peritoneal macrophages and induce a protective immune response. Regulation of these factors significantly prolongs the lifespan of L1210 tumour‐bearing mice and suggests that SEP and APS‐1II can act as drug candidates for the treatment of leukaemia.^20^

**FIGURE 6:**
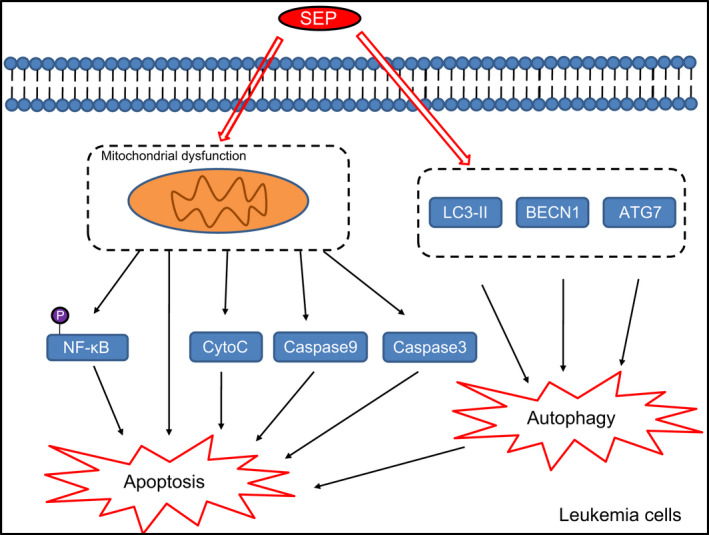
Schematic diagram depicting how SEP‐mediated induction of autophagy leads to activation of apoptosis in AML. Treatment with SEP leads to mitochondrial dysfunction resulting in the activation of caspase‐3 and caspase‐9 as well as the release of CytoC, which triggers apoptosis in leukaemia cells. SEP also induces cell apoptosis by enhancing phosphorylation of NF‐κB and activating NF‐κB signalling pathway. SEP may elevate autophagy‐related protein LC3‐II, ATG7 and BECN1, stimulating autophagy in leukaemia cells and eventually promoting apoptosis

We have shown that SEP promotes the apoptosis of AML cells using a mechanism that requires the NF‐κB signalling pathway (Figure 6). This research has highlighted new markers and therapeutic targets for the treatment of AML. We cannot exclude the possibility that other pathways may directly participate in or interact with the NF‐κB signalling pathway to affect the progression of AML and further study is necessary to provide further insights into these pathways.

## CONFLICT OF INTEREST

The authors confirm that there are no conflicts of interest.

## AUTHOR CONTRIBUTIONS

**Chong Wang:** Conceptualization (equal); Investigation (equal); Methodology (equal); Writing‐review & editing (equal). **Mengya Li:** Data curation (equal); Resources (equal); Software (equal); Writing‐original draft (equal). **Lingling Li:** Validation (equal); Visualization (equal); Writing‐original draft (equal). **Xiaohui Shen:** Methodology (equal); Visualization (equal); Writing‐review & editing (equal). **Yanfang Liu:** Formal analysis (equal); Writing‐original draft (equal). **Shujuan Wang:** Project administration (equal); Supervision (equal); Writing‐review & editing (equal).

## Supporting information

Figure S1

Figure S2

Figure S3

Figure S4

## Data Availability

All data supporting the findings of this study is available within the article.
